# The Eastern Mediterranean Public Health Network: A Resource for Improving Public Health in the Eastern Mediterranean Region

**DOI:** 10.2196/14992

**Published:** 2019-09-06

**Authors:** Mohannad Al Nsour

**Affiliations:** 1 The Eastern Mediterranean Public Health Network Abdallah Ben Abbas St, Building No. 42 Amman Jordan

**Keywords:** field epidemiology, public health, training, research

## Abstract

Countries in the Eastern Mediterranean Region (EMR) face many challenges in terms of improving population health and progressing toward sustainable development goals (SDGs). This paper aims to describe the approach taken by the Eastern Mediterranean Public Health Network (EMPHNET) to help strengthen health systems in the EMR and enable progress toward sustainable development targets, the tools it used, and its achievements. The EMPHNET is a nonprofit organization that has worked to support EMR countries in strengthening their public health systems since its establishment in 2009. The EMPHNET invests in building workforce capacity in applied epidemiology by supporting field epidemiology training programs in more than 10 countries in the EMR, while ensuring country ownership of these programs. The EMPHNET established the Global Health Development (GHD) to maximize support for positive change and SDG progress. As an implementing arm to the EMPHNET, GHD aligns its strategies with national policies and directions. The GHD/EMPHNET works at the regional, national, and subnational levels and tailors solutions for the local context. Over the past years, the EMPHNET succeeded in partnering with over 13 countries and provided technical assistance to leverage country efforts and maximize resource use. The EMPHNET’s Center of Excellence for Applied Epidemiology focuses on building capacity in population health and applied epidemiology. The EMPHNET supports countries in delivering effective public health programs by building capacity and conducting research to prevent and control emerging and reemerging diseases, vaccine-preventable diseases, and noncommunicable diseases. The commitment to the region, together with the increased trust and assertion from the countries, helped GHD/EMPHNET build a strong portfolio, which was made possible by the interconnected effort that continues to nurture and foster better health among people living in the EMR.

## Introduction

The Eastern Mediterranean Region (EMR) embraces countries that vary in economic growth and development level, which significantly affects the health status of the populations in these countries. Regardless of the economic and development level, all EMR countries are facing an increase in the prevalence of noncommunicable diseases [1], with many countries still suffering from the double burden of disease where communicable diseases remain common. Further, EMR countries face many challenges in improving population health and progressing toward sustainable development goals (SDGs) [2]. Progress toward achieving SDGs in several EMR countries is constrained by many shortcomings that hinder improvement in health outcomes such as limited resources, conflict, and political instability [3]. In most countries of the region, programs that address the key health challenges [4] are limited because of limited capacity in public health. In some countries, public health improvement was challenged by emergencies, crises, refugees, and displacement [5].

This paper aims to describe the approach, tools, and achievements of the Eastern Mediterranean Public Health Network (EMPHNET) toward strengthening health systems in the EMR and progressing toward the sustainable development targets.

## The Eastern Mediterranean Public Health Network

The Eastern Mediterranean Public Health Network (EMPHNET) is a nonprofit organization that has worked to support EMR countries in strengthening their public health systems since its establishment in 2009. The EMPHNET invests in building workforce capacity in applied epidemiology by supporting field epidemiology training programs (FETPs) in more than 10 countries in the EMR, while ensuring country ownership of these programs. Increasing demand from several EMR countries to develop their capacities in various areas of public health urged the EMPHNET to expand its efforts in capacity building to address key health priorities and gaps that cover essential public health areas, such as outreach and emergency, bio-risk management, communicable diseases, and health protection and promotion. Its vision is to see people in the EMR lead healthy lives and better welfare. Its mission is to prevent and control diseases, conduct and support operational research for priority public health domains, and strengthen public health programs while working jointly with similar institutions associations, networks, and organizations. EMPHNET’s website [6] displays a lot of information on various tools and approaches, activities, success stories, and achievements of the EMPHNET in the EMR.

## The Eastern Mediterranean Public Health Network’s Efforts Toward Sustainable Development Goals

The EMPHNET believes that change and transformation are key elements for progressing toward the SDGs and that heath is a basic right for all human beings wherever they live. EMPHNET’s work is driven by a deep interest and belief in the importance of achieving universal health coverage, which influence its initiatives and strategies. Therefore, consistent with the SDG’s motto, “Ensure healthy lives and promote well-being for all at all ages” [7], the EMPHNET adopted a transformational vision that guides its role and efforts toward assisting EMR countries strengthen their health systems. Such efforts contributed to meeting key health priorities and gaps that cover essential public health areas, such as health security, emergency preparedness and response, noncommunicable diseases, and communicable diseases. In maximizing its contribution to the SDGs, the EMPHNET acknowledged the role of different factors in influencing health and viewed many of the SDG challenges (such as those related to poverty, hunger, education, inequality, and climate change) as detrimental to achieving a healthy well-being. The EMPHNET also acknowledged the essential role of public health institutions across the globe to achieve the SDG targets. Therefore, the EMPHNET works on building national, regional, and global partnerships under SDG17 [4] as well as secure global health opportunities as an innovative strategy to support EMR countries build robust health systems to meet the SDG challenges.

With this context, the EMPHNET established the Global Health Development (GHD) as an asset to maximize support toward positive change by seizing opportunities for backing up countries in influencing SDG progress. GHD was initiated to advance the work of the EMPHNET by building coordinating mechanisms with Ministries of Health, International Organizations, and other institutions to improve population health outcomes. As an implementing arm to the EMPHNET, GHD aligns its strategies with national policies and directions. Serving as a collaborative platform, the GHD/EMPHNET is dedicated to serve the region by supporting national efforts to promote public health policies, strategic planning, sustainable financing, resource mobilization, public health programs, and other related services.

## The Eastern Mediterranean Public Health Network’s Approach to Strengthening Health Systems

The GHD/EMPHNET adopts a comprehensive approach process to health system strengthening, where it works at different levels to identify and respond to health challenges. The GHD/EMPHNET works at the regional, national, and subnational levels and tailors solutions to the local context. Its approach triggers change by reinforcing knowledge through regional, national, and subnational activities and using a proactive learning approach to bring about effective problem solving and ownership of outcomes. The GHD/EMPHNET’s commitment to support countries bring about change at the frontline level is supported by a bottom-up approach to health system strengthening, where action planning and program management are transformed at the peripheral level, thus producing national-level change.

In supporting countries to address health priorities, the GHD/EMPHNET puts emphasis on engaging and involving stakeholders, which is viewed as an effective strategy for identifying potential opportunities and maximizing the use of resources for rolling out broader actions to improve health outcomes. The GHD/EMPHNET implements operational research to generate information that can link policy to practice and engage in implementation research that contributes to countries’ efforts to reach SDG targets through knowledge synthesis. This approach helps generate evidence from the region to share with the global health sector.

## Global Health Development/Eastern Mediterranean Public Health Network’s Working Areas

Over the past years, the EMPHNET succeeded in partnering with over 13 countries and has worked in close collaboration with a wide range of institutions, partners, and implementers; with different health providers and practitioners; and has provided technical assistance to leverage country efforts and maximize resource use. The deeply rooted collaboration with countries allowed the GHD/EMPHNET to drive and direct opportunities to correspond to priority needs of the countries. Further, the strategy helped expand efforts and tailor new working areas that target essential public health functions in addition to focusing on applied epidemiology, which was the initial onset area of concern.

## Workforce Development

Investing in health workforce development is a key factor in strengthening health systems and supporting progress toward reaching SDG targets. Building a stronger public health workforce improves health system performance by contributing to more effective service delivery. EMPHNET’s Center of Excellence for Applied Epidemiology focuses on building capacity in population health and applied epidemiology. The EMPHNET is committed to support field epidemiology training programs (FETPs) in EMR countries, as these programs are crucial for assuring core epidemiologic competencies. These programs aim at applying scientific methods in the field, such as using epidemiologic methods to investigate health problems or outbreaks or analyzing data gathered through surveillance or other methods to generate evidence for decision makers. Core competencies gained by FETP training add value to meeting the international health regulations by building surveillance capacity and improving efficiency in monitoring disease incidence, prevalence, determinants, coverage, program evaluation, and expenditure data. In addition, FETP training plays a crucial role in strengthening the response to unexpected health problems or events, thus containing and preventing their spread. Integrating applied epidemiology concepts in strengthening a range of services is crucial because skilled field epidemiologists are the core of a robust public health system.

## Public Health Programs

Preventing and controlling communicable and noncommunicable diseases is essential for assuring healthy living and well-being. The EMPHNET supports countries in delivering effective public health programs by building capacity and conducting research to prevent and control emerging and reemerging diseases, vaccine preventable diseases, and noncommunicable diseases. Focus areas under this domain include disease control, outreach and emergency, polio and immunization, health protection and promotion, environmental health, and disease of special concern. Since all health-related SDG targets cover health concerns that countries need to address by developing health programs and related interventions, the GHD/EMPHNET supports EMR countries in developing and strengthening public health programs and in supporting countries translate global initiatives, strategies, and action plans.

## Research and Policy

Monitoring progress and performance is important for assuring progress toward SDG targets. The GHD/EMPHNET supports countries in building robust and reliable information to support translating information into policies. The GHD/EMPHNET works with a range of institutions and builds research experience for field epidemiologists, public health practitioners, and young researchers while highlighting data collection challenges and providing appropriate digital solutions. The GHD/EMPHNET collaborates with academic and nonacademic institutions and similar organizations to generate evidence that can guide policies by focusing on aspects such as operational research, assessments and surveys, secondary data analysis and information generation, public health program monitoring, and evaluation studies.

## Communication and Networking

Effective communication and broad networking are important for assessing gaps and planning and delivering different health programs. The GHD/EMPHNET created a network of public health professionals and experts in the EMR to support program development and service delivery. It also invested in communication and used it to advance information sharing, as it is a key element for assuring robust data systems that countries need to secure to monitor their progress toward SDG targets. EMPHNET’s networking efforts allowed to integrate capacities when assisting countries to explore solutions for meeting the challenges. The GHD/EMPHNET remains committed to network regionally and globally with Ministries of Health, regional, and international organizations as well as private sector and academic institutions to attract opportunities and partnerships that support investment in programs that support implementation of the SDGs.

## The Eastern Mediterranean Public Health Network’s Networking and Knowledge Exchange Platforms

### Internship

The GHD/EMPHNET provides an opportunity for students and fresh graduates to work in and learn from working in a rapidly expanding, multifield public health organization. The internship program allows candidates to work in GHD/EMPHNET’s different work settings, while supporting their own initiatives or projects. The GHD/EMPHNET provide interns with an opportunity to apply professional public health competencies and skills, thus preparing them to work with confidence in real public health settings. Interns at the EMPHNET are mentored by skilled and experienced supervisors who supervise their internship and help them link theory to practice. The GHD/EMPHNET matches the career interest and preference of interns with work situations that allow them to maximize the use of the knowledge they gained during school education. GHD/EMPHNET’s internship program is in high demand, with candidates seeking experience in different areas including infectious diseases, applied epidemiology, health promotion, media, and communication.

### Field Epidemiology Exchange Program

Considering the diversity of public health emergencies, outbreak investigations, and challenges in the EMR, it is of great importance that FETP residents or students studying public health at a university are exposed to a broad range of field experiences. As a leading regional public health network, the GHD/EMPHNET strives to provide an exchange platform to promote applied epidemiology experience sharing between countries, as the levels of health care and system development differ among EMR countries. This exchange program seeks to expand the network of public health experts and field epidemiologists in the region by promoting a diverse field experience. By establishing the FEEP, the GHD/EMPHNET provides the FETP residents and university students an opportunity to join FETP programs in EMR countries for a specific period of time for the purpose of gaining new experiences. Such a program was established in response to a request by FETPs and public health academic institutions in the EMR based on a need to foster public health competencies. In addition to the exchange and network expansion benefits, the FEEP aims to strengthen regional public health emergency response as well as promote coordination between ministries of health in the region. The program will result in an increased number of epidemiologists trained in surveillance and field investigations, who are capable of defining health measures to control disease outbreaks in the region.

### Conferences

The EMPHNET works to link, support, and strengthen public health programs in EMR countries and beyond. The EMPHNET’s biannual regional conference is an opportunity for public health professionals and field epidemiologists working in the EMR to exchange experience and be exposed to new ideas and skills. The conference presents a platform to “showcase” achievements in research, outbreak investigations, assessments, and evaluation. In addition to public health networking, the conference provides the region with a special opportunity for demonstrating progress and innovation in applied epidemiology across countries. To date, the EMPHNET has conducted six regional conferences, where over 1000 scientific research works were presented. The number of submissions and accepted abstracts to the EMPHNET conferences escalated over the years, making such conferences recognizable with a competitive pursue.

### EpiShares

The GHD/EMPHNET developed and launched a networking platform (EpiShares) designed to join public health professionals and experts in a space where they can express thoughts, address concerns, and discuss issues relevant to public health issues. As a unique public health community of practice, EpiShares enables public health professionals and experts to come together to reflect and explore solutions necessary for managing public health challenges and doubtful situations in various settings. Through EpiShares, the GHD/EMPHNET taps into advances in technology to offer an ideal environment that would grant members a chance to ponder into a common space where they can examine interests of other colleagues, identify colleagues with mutual interests, and seek the advice or opinion of experts. The GHD is enabling members of EpiShares to share articles, opportunities, tools, training resources, and other essential elements needed to enhance their performance and expand their scope and perspectives. EpiShares will be the space that will embrace its members’ credentials and grow into a powerful public health sphere whereby experts can be accessed and contacted for building initiatives, programs, or schemes.

## The Eastern Mediterranean Public Health Network’s Public Health Forum

Acting as a forum for public health professionals in EMR since 2009, the EMPHNET officially established a Public Health Forum (PHF) in 2018 to serve as a platform that brings together public health professionals to discuss and get involved in issues that affect public health practice. Modelled to support improvements to public health, the EMPHNET-PHF is involved in networking between different health sectors to improve public health and contributing to debates and discussions that can shape or influence public health policy. The EMPHNET-PHF brings different stakeholders and professionals together while working actively to promote dialogue that influences policy making. This forum works by holding regular meetings to discuss concerns and issues that challenge public health.

## Challenges

One of the main challenges facing the EMPHNET is the that many of its activities are carried out in countries that are politically unstable or in crisis. These conditions might limit the movement of staff to attend the EMPHNET workshops and events. However, there are several enabling factors that allow the EMPHNET to affect change and contribute to better performing heath systems in EMR countries. These factors stem from internal and external strengths and contextual factors that help maximize the effect and impact of opportunities sought to address challenge and from collaboration with governments and employment of a network of experts and professionals to conduct research and produce training material, toolkits, and guidelines.

## Conclusions

Since its establishment in 2009, the EMPHNET implemented activities to support strengthening of public health systems in EMR countries. Through collaborative efforts with partners and cooperation with ministries of health, the GHD/EMPHNET managed to reach a status that is recognized in the global arena. The commitment to the region together with the increased trust and assertion from the countries helped the GHD/EMPHNET build a strong portfolio made possible with interconnected effort that continues to nurture and foster merit for better health among people living in the EMR.

## Abbreviations

EMPHNET: Eastern Mediterranean Public Health Network
EMR: Eastern Mediterranean Region
FETP: field epidemiology training programs
GHD: Global Health Development
PHF: Public Health Forum
SDG: sustainable development goal
